# Comparative study of virulence factors among methicillin resistant *Staphylococcus aureus* clinical isolates

**DOI:** 10.1186/s12879-018-3457-2

**Published:** 2018-11-13

**Authors:** Ons Haddad, Abderrahmen Merghni, Aida Elargoubi, Hajer Rhim, Yosr Kadri, Maha Mastouri

**Affiliations:** 1 Laboratoire de Microbiologie, CHU Fatouma Bourguiba de Monastir, Monastir, Tunisie; 2 Laboratoire des Maladies Transmissible et Substances Biologiquement Actives, LR99ES27, Faculté de Pharmacie de Monastir, Université de Monastir, Monastir, Tunisie

**Keywords:** *Staphylococcus aureus*, methicillin, biofilm, virulence, adhesion molecules

## Abstract

**Background:**

Methicillin resistant *Staphylococcus aureus* (MRSA) is recognized worldwide as a leading cause of hospital and community infections. Biofilm formation by MRSA is an extremely important virulence factor to be understood. Our aim was to establish phenotypic and genotypic characterization of virulence factors among 43 MRSA clinical isolates in a Tunisian hospital.

**Methods:**

We investigated enzymatic profiles, biofilm production and prevalences of genes encoding intracellular adhesion molecules (*icaA* and *icaD*), Microbial Surface Components Recognizing Adhesive Matrix Molecules genes (*fnbA*, *fnbB* and *cna*) and exoenzymes genes (*geh*, *sspA* and *sspB*).

**Results:**

Our findings revealed that caseinase, gelatinase, lipase and lecithinase activities were detected in 100%, 100%, 76.6% and 93.3% of cases respectively. This study showed that 23 strains (76.7%) were slime producers on Congo red medium. Furthermore, 46.5% and 53.5% of isolates were respectively highly and moderately biofilm-forming on polystyrene. Significant association was found between both biofilm tests. PCR detection showed that 74.4%, 18.6%, 69.8%, 65.1% and 74.4% of isolates harbored *fnbA*, *fnbB*, *icaA*, *icaD* and *cna* genes respectively. In addition, 34.9%, 18.6% and 30.2% of MRSA strains were found positive for *sspA*, *sspB* and *geh* genes respectively. Further, statistical data showed that the presence of the *fnbA* and *fnbB* genes was significantly associated with a high biofilm production on polystyrene. However, no statistical association was observed for the *icaA*, *icaD* and *cna* genes.

**Conclusions:**

This study indicates that the detection of *fnbA* and *fnbB* contributing to the first step of biofilm formation has been predictable of high biofilm production. As studied factors contribute to MRSA virulence, this research could be of value in orienting towards the development of new preventive and therapeutic measures.

## Introduction

Methicillin resistant *Staphylococcus aureus* (MRSA) is recognized worldwide as an important bacterial pathogen causing a wide range of infections ranging from skin and soft tissue lesions to lethal infections (osteomyelitis, endocarditis, pneumonia and septicaemia). It is regarded as a major world health threat with a substantial increase in morbidity and mortality [1]. In addition to resistance, the pathogenicity of MRSA is an extremely important feature to be understood. The pathogenesis of this bacterium depends on a combination of extracellular factors and biofilm forming ability [2]. The adherence of *S. aureus* to biotic and abiotic surfaces stage is mediated by a protein family of staphylococcal Microbial Surface Components Recognizing Adhesive Matrix Molecules (MSCRAMMs). Whereas the cell aggregation is led by the synthesis of polysaccharide intercellular adhesin (PIA) molecule encoded by the intracellular adhesion (*ica*) [3–6].

Our aim was to establish a phenotypic characterization of virulence factors among MRSA clinical isolates in a Tunisian Hospital, to determine prevalences of genes encoding intracellular adhesion molecules (*icaA* and *icaD*), MSCRAMMs genes (*fnbA*, *fnbB*, *cna*) and exoenzymes genes (*geh*, *sspA* and *sspB*), and to investigate the involvement of these genes in biofilm forming ability.

## Materials and methods

### Bacterial strains

A total of 43 non-redundant MRSA clinical isolates were obtained from the Laboratory of Microbiology of the University hospital of Monastir-Tunisia. These strains were obtained from bacteriological samples in hospitalized patients and/or consultants at the University Hospital of Monastir-Tunisia during the period (January 2016 - June 2016). Two reference strains (*S. aureus* ATCC 6538 and ATCC 43300) were used as controls. Bacterial identification was performed using Gram staining, catalase test, tube coagulase, DNase agar, mannitol salt agar and API ID 20 STAPH galleries (bio-Merieux, France). To confirm the identity of the isolate as *S. aureus*, the *Sa442* gene was amplified by a PCR-based method. Extraction of genomic DNA was performed using a standard phenolechloroform technique. The PCR mixture (25 μl) for all genes contained 1 mM forward and reverse primers, dNTP mix (100 mM each of dATP, dCTP, dGTP and dTTP), 1 U of Go Taq DNA polymerase (Promega), 5 ml green Go Taq buffer (5X), and DNA template (50 ng). PCR conditions were the following: an initial temperature of 96°C (3min), followed by denaturation at 95°C (1min), annealing at 55°C (30s), elongation at 72°C (3min), and a final elongation step at 72°C (4min). Amplicons of the expected size (108 bp) were obtained. Methicillin resistance was confirmed using a cefoxitin disk (30 μg) on Mueller-Hinton agar plates (Bio-Rad, France) as recommended by the French Society of Microbiology and by the European Committee on Antimicrobial Susceptibility Testing. The *mecA* gene was detected by PCR. PCR conditions were the following : an initial temperature of 95°C (1min), denaturation at 95°C (15s), annealing at 45°C (15 s), and elongation at 72°C (30s), with a final extension at 72°C (4min). Amplicons of the expected size (162 bp) were obtained [7]. PCR primers were chosen as listed in Table 1.

**Table 1:** List of primers used for bacterial identification and detection of MSCRAMMs genes (*fnbA*, *fnbB* and *cna*), biofim control genes (*icaA* and *icaD*) and exoenzymes genes (*sspA*, *sspB* and *geh*)

*Target gene*	Sequence (5’-3’)	aT (°C)	Amplified fragment	Amplicon size (bp)	Reference
*Sa442*	5’-AATCTTTGTCGGTACACGATATTCTTCACG-3’ 5’-CGTAATGAGATTTCAGTAAATACAACA-3’	55	Sa442 protein	108	[7]
*mecA*	5’-ACCAGATTACAACTTCACCAGG-3’ 5’-CCACTTCATATCTTGTAACG-3’	45	Penicillin binding protein 2a	162	[7]
*ica*A	5’-ACACTTGCTGGCGCAGTCAA-3’ 5’-TCTGGAACCAACATCCAACA-3’	55	Intercellular adhesion A	188	[24]
*ica*D	5’-ATGGTCAAGCCCAGACAGAG-3’ 5’-AGTATTTTCAATGTTTAAAGCAA-3’	55	Intercellular adhesion D	198	[24]
*Can*	5’-GTCAAGCAGTTATTAACACCAGAC-3’ 5’-AATCAGTAATTGCACTTTGTCCACTG-3’	62	Collagen adhesion	192	[36]
*fnb*A	5’-CATAAATTGGGAGCAGCATCA-3’ 5’-ATCAGCAGCTGAATTCCCATT-3’	62	Fibronectin binding protein A	191	[36]
*fnb*B	5’-GTAACAGCTAATGGTCGAATTGATACT-3’ 5’-CAAGTTCGATAGGAGTACTATGTTC-3’	62	Fibronectin binding protein B	201	[36]
*sspA*	5’-GACAACAGCGACACTTGT 3’ 3’-AGTATCTTTACCTACAACTACA-5’	45	Serine protease	292	[40]
*sspB*	5’-TGAAGAAGATGGCAAAGTTAG-3’ 3’-TTGAGATACACTTTGTGCAAG-5’	47	Cysteine protease	493	[40]
*Geh*	5’-GCACAAGCCTCGG -3’ 3’-GACGGGGGTGTAG-5’	40	Lipase	473	[45]

*aT* annealing temperature

Amplified PCR products were analyzed on 2% (wt/v) agarose gel stained with ethidium bromide (0.5 μg ml^-1^), visualized under ultraviolet transillumination and photographed using Gel Doc XR apparatus (Biorad, USA).

### Characterization of the enzymatic activity

The ability to produce hydrolytic enzymes was determined after inoculation of cultures on TSA-1 media (Biorad) supplemented with: 1% (wt/vol) skim milk for caseinase; 1% (wt/vol) gelatin for gelatinase; Tween 80 for lipase and 5% (vol/vol) egg yolk for lecithinase. The presence of a clear halo around the colonies indicates the presence of the hydrolytic enzyme. The haemolytic activity was evaluated on bacteriological agar supplemented with 5% sheep’s blood [8].

### Phenotypic determination of slime production

Phenotypic qualitative characterization of slime producing strains was carried out by the cultivation of the isolates on Congo red agar (CRA) plate made by mixing 36 g of sucrose (Sigma Chemical Company, St. Louis, MO) with 0.8 Congo red in 1L of brain heart infusion agar (Biorad, USA) as previously described [9]. The strains were incubated at 37°C for 24 hours under aerobic conditions followed by subsequent storage at room temperature. The Congo red dye interacts directly with certain bacterial polysaccharides forming a slime and giving black colonies in contrast to the non-producing colonies which remain red. After 48-72 hours, the results were interpreted as follows: strains producing intensive black, black, and reddish black colonies with a rough, dry, and crystalline consistency were classified as slime producers, whereas red and Bordeaux red with smooth colonies were considered to be non slime producers [9].

### Semi-quantitative adherence assay

Biofilm production by MRSA strains, grown in Brain Heart Infusion with 1% glucose, was quantified using a semi-quantitative adherence assay on 96-well tissue culture plates as described previously [10]. Adherent bacteria were fixed with 95% ethanol and stained with 100 mL of 1% crystal violet (Merck, France) for 5 min. The microplates were air-dried and the optical density of each well was measured at 570 nm (OD570) using an automated Multiskan reader (GIO. DE VITA E C, ome, Italy). This assay was performed for each strain in triplicate. The background was determined by using noninoculated media as a control. The cut-off value (ODC) of noninoculated media at an optical density of 570 nm (OD570) was considered the deadline point to define biofilm quantities [cut-off (ODC) = mean OD + 3 standard deviation (SD) of negative control]. Biofilm formation was interpreted as highly positive (OD570> = 4 * ODC), moderately positive (2 * ODC <= OD570 <4 * ODC), weakly positive (ODC <= OD570 <2 * ODC) or negative (OD570 < ODC). These criteria were established by Stepanovic et al. [11].

### Detection of *icaA*, *icaD*, *fnbA*, *fnbB*, *cna*, *sspA*, *sspB* and *geh* genes

PCR primers were chosen as listed in Table 1. Amplifications of *icaA*, *icaD*, *fnbA*, *fnbB*, *cna*, *sspA*, *sspB* and *geh* genes were performed according to the following cycle conditions: an initial denaturation at 94°C for 5 min was followed by 30 cycles of denaturation at 94°C for 30s, annealing for 30s at temperature determined for each gene as described in Table 1 and elongation at 72°C for 45s, followed by 10 min of final extension at 72°C.

### Statistical analysis

The data from this study were captured, recorded and analyzed by SPSS 17.0 software. The non-parametric Mann Whitney U test was used to compare biofilm production assays (Congo red test and polystyrene adherence assay). The same statistical test was used to evaluate correlations of MSCRAMMs genes (*fnbA*, *fnbB* and *cna*), biofilm production control genes (*icaA* and *icaD*) and exoenzymes genes (*sspA*, *sspB* and *geh*) to the level of biofilm production on polystyrene. All factors with p values of less than 0.20 were included in multivariate ordinal logistic regression analysis. *P* values of less than 0.05 were considered to indicate significant difference.

## Results

### Distribution of isolates

A total of 43 MRSA strains were collected (i.e. a rate of 21.4% of all S. aureus isolates in the laboratory of Microbiology at the University Hospital of Monastir-Tunisia during the same period). Samples were gathered from different organs and systems: pus (58.1%), blood (25.6%), respiratory samples (11.6%) and catheters (4.7%). They were collected from patients admitted in surgery units (44.2%), intensive care units (25.6%), medical units (13.9%), Pediatrics/Neonatology (9.3%) and Gynecology (7.0%).

### Characterization of the enzymatic activity

Our results showed that 51.1% and 48.9% of isolates were respectively beta and alpha-hemolytic. All isolates were protease producers (caseinase and gelatinase). Lipase and lecithinase secretion were found in 93.0% and 79.1% of cases respectively (Table 2).

**Table 2:** Exoenzymes production and hemolysis of studied strains

Strains	Exoenzymes expression				Hemolysis type
	Lecithinase	Lipase	Caseinase	Gelatinase	
ATCC6538	+	+	+	+	Beta
1	+	+	+	+	Alpha
2	+	+	+	+	Alpha
3	+	+	+	+	Alpha
4	+	+	+	+	Alpha
5	+	+	+	+	Beta
6	+	+	+	+	Beta
7	+	+	+	+	Beta
8	-	-	+	+	Beta
9	+	+	+	+	Alpha
10	+	+	+	+	Beta
11	+	+	+	+	Alpha
12	+	+	+	+	Beta
13	+	+	+	+	Beta
14	+	-	+	+	Alpha
15	+	+	+	+	Alpha
16	+	-	+	+	Beta
17	+	-	+	+	Beta
18	+	+	+	+	Alpha
19	+	+	+	+	Beta
20	+	+	+	+	Alpha
21	+	+	+	+	Beta
22	+	+	+	+	Alpha
23	-	-	+	+	Beta
24	+	+	+	+	Alpha
25	+	+	+	+	Alpha
26	+	+	+	+	Beta
27	+	+	+	+	Beta
28	+	+	+	+	Alpha
29	+	+	+	+	Alpha
30	-	-	+	+	Beta
31	+	+	+	+	Beta
32	+	+	+	+	Alpha
33	+	+	+	+	Alpha
34	+	+	+	+	Alpha
35	+	+	+	+	Beta
36	+	-	+	+	Alpha
37	+	+	+	+	Beta
38	+	+	+	+	Beta
39	+	+	+	+	Alpha
40	+	-	+	+	Beta
41	+	+	+	+	Alpha
42	+	+	+	+	Beta
43	+	-	+	+	Beta
% expression	93,0%	79,1%	100%	100%	48.9%(alpha);51.1%(beta)

### Phenotypic determination of slime production

Out of 43 MRSA isolates, 30 strains (69.8%) were slime producers on the CRA plate. They were black or reddish black color colony producers. Slime production was noted in 16 out of 25 strains isolated from suppurations and in 9 out of 11 blood culture isolates (Table 3).

**Table 3:** Slime production and adherence assay of methicillin resistant *Staphylococcus aureus* isolates

Strains	Biofilm phenotype (CRA)	OD570 ± SD	Adherence state
ATCC 6538	S+	2.90±0.05	highly positive
ATCC 43300	S+	0.71±0.15	highly positive
1	S-	0.19±0.05	moderately positive
2	S-	0.12±0.03	moderately positive
3	S+	0.13±0.02	moderately positive
4	S+	0.59±0.11	highly positive
5	S+	0.46±0.02	highly positive
6	S+	0.26±0.01	highly positive
7	S+	0.22±0.08	moderately positive
8	S+	0.42±0.01	highly positive
9	S-	0.15±0.02	moderately positive
10	S+	0.80±0.03	highly positive
11	S+	2.96±0.08	highly positive
12	S-	0.15±0.04	moderately positive
13	S-	0.15±0.07	moderately positive
14	S+	1.77±0.02	highly positive
15	S+	0.22±0.01	moderately positive
16	S+	0.17±0.03	moderately positive
17	S+	2.62±0.01	highly positive
18	S+	0.24±0.02	highly positive
19	S+	0.13±0.05	moderately positive
20	S+	0.62±0.03	highly positive
21	S+	0.12±0.03	moderately positive
22	S+	0.21±0.39	moderately positive
23	S+	0.14±0.06	moderately positive
24	S+	0.14±0.01	moderately positive
25	S+	0.14±0.01	moderately positive
26	S+	1.00±0.12	highly positive
27	S-	0.18±0.13	moderately positive
28	S+	0.16±0.30	moderately positive
29	S-	0.26±0.60	highly positive
30	S+	0.14±0.02	moderately positive
31	S-	0.20±0.03	moderately positive
32	S+	1.51±0.08	highly positive
33	S+	0.41±0.09	highly positive
34	S+	0.40±0.07	highly positive
35	S+	0.27±0.06	highly positive
36	S+	0.31±0.04	highly positive
37	S+	0.23±0.02	highly positive
38	S+	0.37±0.05	highly positive
39	S-	0.18±0.02	moderately positive
40	S-	0.16±0.03	moderately positive
41	S-	0.22±0.04	moderately positive
42	S-	0.25±0.05	highly positive
43	S-	0.12±0.03	moderately positive

### Semi-quantitative adherence assay

The results of OD570 presented in Table 3, showed that 46.5% and 53.5% strains were respectively highly and moderately biofilm-formers on polystyrene. No isolate was weakly or non-biofilm producer on polystyrene. We detected 5/11 blood culture isolates and 10/25 pus isolates considered as highly biofilm formers. Both reference strains *S. aureus* ATCC 6538 and ATCC 43300 were highly biofilm-forming on polystyrene.

### Detection by PCR of icaA, icaD, fnbA, fnbB, cna, sspA, sspB and geh genes

The *icaA* and the *icaD* genes were present in 69.8% and 65.1% of MRSA isolates respectively. A concomitant presence of both genes was detected in 55.8% of the strains. Our results revealed that 74.4% and 18.6% of isolates respectively harbored the *fnbA* and *fnbB* genes. The *cna* gene coding for adhesin to collagen was detected in 32 strains (74.4%). Among the tested strains, 15 (34.9%), 8 (18.6%) and 13 (30.2%) were positive for *sspA*, *sspB* and *geh* genes.

### Statistical analysis

#### Association between Congo red phenotypic test and adherence assay

Out of 20 highly-biofilm producing isolates, 18 showed a positive slime phenotype on Congo red agar. The nonparametric test of Mann Whitney U demonstrated that high biofilm production on polystyrene was significantly correlated with a positive Congo red phenotypic test (*p* = 0.017).

#### Association between biofilm production on polystyrene and biofilm related genes

Table 4 shows prevalence rates of MSCRAMMs genes (*fnbA*, *fnbB* and *cna*) and biofilm production control genes (*icaA* and *icaD*) depending on biofilm production. Univariate analysis followed by multivariate analysis showed that *fnbA* and *fnbB* genes were significantly associated with high biofilm production on polystyrene. However, no difference was observed in prevalence rates of *icaA*, *icaD* and *cna* genes between highly and moderately biofilm producing groups.

**Table 4:** Association between biofilm production on polystyrene and biofilm related genes

Genes		Moderately bioflm producers (*n*=23)	Highly biofilm producers (*n*=20)	P (univariate analysis)	P (multivariate analysis)
MSCRAMMs genes	*fnbA+*	14/23	18/20	0,001^a^	0,040
	*fnbB+*	2/23	6/20	0,002^a^	0,003
	*cna+*	15/23	17/20	0,154^a^	0,981
Biofilm production control genes	*icaA+*	13/23	17/20	0,143^a^	0,661
	*icaD+*	14/23	13/20	0,546	-

^a^ Factor studied in mutivariate analysis

#### Association between biofilm production on polystyrene and exoenzymes genes

Table 5 details the prevalence rates of genes encoding serine protease (*sspA*), cysteine protease (*sspB*) and lipase (*geh*) in moderate and high biofilm-producing groups. Analysis showed that the presence of *sspB* gene was significantly associated with high biofilm production on polystyrene (p=0.003). However, no differences were found in prevalence rates of *sspA* and *geh* genes between both groups (Table 5).

**Table 5:** Association between biofilm production on polystyrene and exoenzymes genes

Exoenzymes genes	Moderately bioflm producers (*n*=23)	Highly biofilm producers (*n*=20)	P (univariate analysis)	P (bivariate analysis)
*geh+*	8/23	5/20	0,791	-
*sspA+*	6/23	9/20	0,117^a^	0,223
*sspB+*	1/23	5/20	0,013^a^	0,003

^a^ Factor studied in bivariate analysis

## Discussion

*S. aureus* has emerged in recent decades as one of the leading causes of hospital and community infections [12]. Its virulence is a multifactorial process requiring the use of a variety of components regulated in a coordinated manner [2]. In this study, we noted that all isolates were alpha or beta-hemolytic. This finding agrees with the results of Barretti et al [13]. Hemolysin-α has pore-forming and pro-inflammatory properties. It binds to a membrane receptor and disrupts the integrity of host cells [14]. As for β-toxin, it is known to be the “hot-cold” hemolysin with a sphingomyelinase and a biofilm ligase activities [15–17].

Protease, lipase and lecithinase secretion were detected in most tested strains. Similar results were found by many reports [13, 18, 19]. *S. aureus* uses proteolytic and lipolytic exoenzymes to invade, damage the host tissue components and even spread to other sites [20]. They also protect against the innate immune system and are key mediators of secreted and cell wall-associated virulence determinant stability [21].

Biofilm formation is a major virulence factor. It provides inter-bacterial contact, accumulation of bacteria in superimposed layers, protection against the host immunity and acquisition of significant multiresistance [22, 23]. Our study showed that the majority of strains were slime producers on CRA. Several reports noted lower frequencies [13, 24–30]. In addition, all isolates were highly or moderately biofilm-forming on polystyrene. According to other studies, adhesion capacity was weaker [1, 4, 9, 10, 27, 28, 31–34]. This variability in adhesion capacity between MRSA strains can be explained by inter-strain variability of surface associated proteins and of biofilm production regulatory genes [23]. Significant association between biofilm production on polystyrene and Congo red test in our study highlights the performance of this non-costly phenotypic test to detect potential virulent *S. aureus* clinical isolates. Similarly, previous reports showed a high consistency between both tests [28, 30, 31].

Biofilm formation in *S. aureus* isolates occurs through the polysaccharide intercellular adhesin (PIA) as well as the MSCRAMMs. These structures provide initial binding of *S. aureus* to host tissues and biomaterials [29]. After the step of adhesion and colonization of host cells and mucous membranes, a phase of tissue invasion occurs. It was demonstrated that fibronectin binding proteins FnbA and FnbB are involved in bacterial invasion of the endothelial cells in vivo and in vitro [35]. Prevalences of *fnbA* and *fnbB* genes were higher in other studies [36, 37]. Moreover, the intracellular adhesion (*ica*) operon is essential for the control of biofilm production. The synthesis of polymer matrix exopolysaccharides is monitored by the *icaADBC* operon, which encodes three membrane proteins (IcaA, IcaD, and IcaC) with enzymatic activity and one extracellular protein (IcaB). The PIA, encoded by this operon, plays also an important role in adhesion to epithelial cells and allows escaping the immune system of the host. Frequencies of *icaA* and *icaD* genes were also higher in several previous studies [4, 24, 26–28, 35, 37]. Our results showed that *icaA* and *icaD* genes were not present in all highly biofilm formers. This discrepancy between biofilm phenotype and genotype could be due to the fact that *ica* expression is dependent on environmental conditions [38].

Adhesion to collagen plays an important role in the pathogenesis induced by *S. aureus* [36]. The collagen binding protein Cna is the second most important adhesion molecule. Furthermore, the recombinant Cna can even be designed as an effective vaccine component and antibodies raised against Cna are protective in a mouse model of *S. aureus* induced septic death [39]. Most of studied isolates were positive for *cna* gene. Other reports revealed lower rates [4, 35, 37, 40].

The complexity of bacterial tools used for cell adherence and invasion ranges from single monomeric proteins to intricate multimeric macromolecules that perform highly sophisticated functions [41]. In contrast with this study, *sspA*, *sspB* and *geh* genes were harbored by all strains of another report [40].

- With regard to the complexity and the variability of the biofilm phenotype, genetic studies identified numerous genes involved in biofilm formation. However, their relative importance is still unclear [42]. In the light of this, our multivariate analysis showed that the presence of *fnbA* and *fnbB* genes was significantly associated with a high biofilm production on polystyrene. This consolidates the alleged role of these genes in biofilm formation. Comparably, some studies suggested that detection of some adhesion factors is more practical for the prediction of biofilm formation [4, 31, 43]. Other reports have shown the decisive role of the *ica* gene [32, 33]. However, no statistical difference was found in the distribution of genes by some researchs [28, 31]. Such investigations aim to find new attractive targets for antivirulence therapy by overcoming biofilm formation [31].

We also found a significant association between the presence of *sspB* gene and biofilm production. In fact, a number of soluble extracellular proteins can affect biofilm formation. The relationship between staphylococcal enzymes activities and biofilm formation was reported by some studies. However, it remains to be elucidated and merits further investigation [44].

## Conclusion

In summary, most of our MRSA isolates were highly biofilm producers with elevated prevalences of some MSCRAMMs genes and biofilm production control genes. The present study indicates that the detection of the adhesion factors (*fnbA* and *fnbB* genes) contributing to the first step of biofilm formation can be used as a biofilm formation marker in *S. aureus*. Further in-depth research could be of value in the development of new preventive and therapeutic measures of staphylococcal infections.

## Abbreviations

ATCC: American Type Culture Collection
Cna: Collagen binding protein
CRA: Congo red agar
DNA: Deoxyribonucleic acid
Fnb: Fibronectin binding protein
Ica: Intracellular adhesion
MRSA: Methicillin resistant *Staphylococcus aureus*
MSCRAMMs: Microbial Surface Components Recognizing Adhesive Matrix Molecules
OD: Optical density
PCR: Polymerase chain reaction
PIA: Polysaccharide intercellular adhesion
TSA: Trypticase soja agar

## References

[1] Naicker PR, Karayem K, Hoek KG, Harvey J, Wasserman E (2016). Biofilm formation in invasive *Staphylococcus aureus* isolates is associated with the clonal lineage. Microb Pathog.

[2] Dinges MM, Orwin PM, Schlievert PM (2000). Exotoxins of *Staphylococcus aureus*. Clin Microbiol Rev.

[3] Donlan RM (2002). Biofilms: microbial life on surfaces. Emerg Infect Dis.

[4] Khoramian B, Jabalameli F, Niasari-Naslaji A, Taherikalani M, Emaneini M (2015). Comparison of virulence factors and biofilm formation among *Staphylococcus aureus* strains isolated from human and bovine infections. Microb Pathog..

[5] Abraham NM, Jefferson KK (2010). A low molecular weight component of serum inhibits biofilm formation in *Staphylococcus aureus*. Microb Pathog.

[6] Dobinsky S, Kiel K, Rohde H, Bartscht K, Knobloch JK, Horstkotte MA (2003). Glucose-related dissociation between icaADBC transcription and biofilm expression by *Staphylococcus epidermidis*: evidence for an additional factor required for polysaccharide intercellular adhesin synthesis. J Bacteriol.

[7] Milheiriço C, Oliveira DC, de Lencastre H (2007). Multiplex PCR strategy for subtyping the staphylococcal cassette chromosome mec type IV in methicillin-resistant *Staphylococcus aureus*: ‘SCCmec IV multiplex’. J Antimicrob Chemother.

[8] Stulik L, Malafa S, Hudcova J, Rouha H, Henics BZ, Craven DE (2014). α- Hemolysin activity of methicillin-susceptible *Staphylococcus aureus* predicts ventilator-associated pneumonia. Am J Respir Crit Care Med.

[9] Kouidhi B, Zmantar T, Hentati H, Bakhrouf A (2010). Cell surface hydrophobicity, formation, adhesives properties and molecular detection of adhesins genes in *Staphylococcus aureus* associated to dental caries. Microb Pathog.

[10] Manago K, Nishi J, Wakimoto N, Miyanohara H, Sarantuya J (2006). Biofilm formation by and accessory gene regulator typing of methicilin-resistant *Staphylococcus aureus* strains recovered from patient with nosocomial infections. Infect Control Hosp Epidemiol.

[11] Stepanovic S, Vukovic D, Dakic I, Savic B, Svabic-Vlahovic M (2000). A modified microtiter-plate test for quantification of staphylococcal biofilm formation. J Microbiol Methods.

[12] Gould I (2006). Costs of hospital-acquired methicillin-resistant *Staphylococcus aureus* (MRSA) and its control. Int J Antimicrob Agents.

[13] Barretti P, Montelli AC, Batalha JE, Caramori JC, Cunha Mde L. The role of virulence factors in the outcome of staphylococcal peritonitis in CAPD patients. BMC Infect Dis. 2009;9(212) 10.1186/1471-2334-9-212. 10.1186/1471-2334-9-212 PMC2807432 20028509

[14] Inoshima N, Wang Y, Wardenburg JB (2012). Genetic requirement for ADAM10 in severe *Staphylococcus aureus* skin infection. J Invest Dermatol.

[15] Salgado-Pabón W, Herrera A, Vu BG, Stach CS, Merriman JA (2014). *Staphylococcus aureus* β-toxin production is common in strains with the β-toxin gene inactivatedby bacteriophage. J Infect Dis.

[16] Diep BA, Carleton HA, Chang RF, Sensabaugh GF, Perdreau-Remington F (2006). Roles of 34 virulence genes in the evolution of hospital- and community-associated strains of methicillin-resistant *Staphylococcus aureus*. J Infect Dis.

[17] Huseby MJ, Kruse AC, Digre J (2010). Beta toxin catalyzes formation of nucleoprotein matrix in staphylococcal biofilms. Proc Natl Acad Sci USA.

[18] Wu PZ, Zhu H, Thakur A, Willcox MD (1999). Comparison of potential pathogenic traits of staphylococci that may contribute to corneal ulceration and inflammation. Aust N Z J Ophthalmol.

[19] Lakshmi HP, Prasad UV, Yeswanth S, Swarupa V, Prasad OH, Narasu ML (2013). Molecular characterization of α-amylase from *Staphylococcus aureus*. Bioinformation.

[20] Kolar SL, Ibarra JA, Rivera FE, Mootz JM, Davenport JE (2013). Extracellular proteases are key mediators of *Staphylococcus aureus* virulence via the global modulation of virulence-determinant stability. Microbiologyopen.

[21] Donlan RM, Costerton JW (2002). Biofilms: survival mechanisms of clinically relevant microorganisms. Clin Microbiol Rev.

[22] Pozzi C, Waters E, Rudkin J, Schaeffer C, Lohan A, Tong P (2012). Methicillin Resistance Alters the Biofilm Phenotype and Attenuates Virulence in *Staphylococcus aureus* Device-Associated Infections. PLoS Pathog.

[23] Mulcahy ME, Geoghegan JA, Monk IR, O’Keeffe KM, Walsh EJ, Foster TJ (2012). Nasal colonisation by *Staphylococcus aureus* depends upon clumping factor B binding to the squamous epithelial cell envelope protein loricrin. PLoS Pathog.

[24] El-Mahallawy HA, Loutfy SA, El-Wakil M, El-Al AK, Morcos H (2009). Clinical implications of icaA and icaD genes in coagulase negative staphylococci and *Staphylococcus aureus* bacteremia in febrile neutropenic pediatric cancer patients. Pediatr Blood Cancer.

[25] Elkhatib WF, Khairalla AS, Ashour HM (2014). Evaluation of Different Microtiter Plate-Based Methods for the Quantitative Assessment of *Staphylococcus aureus* Biofilms. Future Microbiol.

[26] Arciola CR, Baldassarri L, Montanaro L (2001). Presence of icaA and icaD genes and slime production in a collection of staphylococcal strains from catheter-associated infections. J Clin Microbiol.

[27] Lindsay JA, Moore CE, Day NP, Peacock SJ, Witney AA, Stabler RA (2006). Microarrays reveal that each of the ten dominant lineages of *Staphylococcus aureus* has a unique combination of surface-associated and regulatory genes. J Bacteriol.

[28] Grinholc M, Wegrzyn G, Kurlenda J (2007). Evaluation of biofilm production and prevalence of the icaD gene in methicillin-resistant and methicillin-susceptible *Staphylococcus aureus* strains isolated from patients with nosocomial infections and carriers. FEMS Immunol Med Microbiol.

[29] Jain A, Agarwal A (2009). Biofilm production, a marker of pathogenic potential of colonizing and commensal staphylococci. J Microbiol Methods.

[30] Arciola CR, Campoccia D, Baldassarri L, Donati ME, Pirini V, Gamberini S (2006). Detection of biofilm formation in *Staphylococcus epidermidis* from implant infections. Comparison of a PCR-method that recognizes the presence of ica genes with two classic phenotypic methods. J Biomed Mater Res A.

[31] Cha JO, Yoo JI, Yoo JS, Chung HS, Park SH, Kim HS (2013). Investigation of Biofilm Formation and its Association with the Molecular and Clinical Characteristics of Methicillin-resistant *Staphylococcus aureus*. Osong Public Health Res Perspect.

[32] Nourbakhsh F, Namvar AE. Detection of genes involved in biofilm formation in *Staphylococcus aureus* isolates. GMS Hyg Infect Control. 2016;22(11) 10.3205/dgkh000267. 10.3205/dgkh000267 PMC4804124 27303652

[33] Szczuka E, Urbańska K, Pietryka M, Kaznowski A (2013). Biofilm density and detection of biofilm-producing genes in methicillin-resistant *Staphylococcus aureus* strains. Folia Microbiol.

[34] Namvar AE, Asghari B, Ezzatifar F, Azizi G, Lari AR. Detection of the intercellular adhesion gene cluster (ica) in clinical *Staphylococcus aureus* isolates. GMS Hyg Infect Control. 2013;29(8) 10.3205/dgkh000203. 10.3205/dgkh000203 PMC3746602 23967389

[35] Peacock S, Moore C, Justice A, Kantazanou M, Story L, Mackie K (2002). Virulent combinations of adhesin and toxin genes in natural populations of *Staphyloccus aureus*. Infect Immun.

[36] Arciola CR, Campoccia D, Gamberini S, Baldassarri L, Montanaro L (2005). Prevalence of cna, fnbA and fnbB adhesin genes among *Staphylococcus aureus* isolates from orthopedic infections associated to different types of implant. FEMS Microbiol Lett.

[37] Rahimi F, Katouli M, Karimi S (2016). Biofilm production among methicillin resistant *Staphylococcus aureus* strains isolated from catheterized patients with urinary tract infection. Microb Pathog.

[38] Zmantar T, Chaieb K, Makni H, Miladi H, Abdallah FB, Mahdouani K (2018). Detection by PCR of adhesins genes and slime production in clinical *Staphylococcus aureus*. J Basic Microbiol.

[39] Madani A, Garakani K, Mofrad MRK (2017). Molecular mechanics of *Staphylococcus aureus* adhesin, CNA, and the inhibition of bacterial adhesion by stretching collagen. PLoS One.

[40] Karlsson A, Arvidson S (2002). Variation in extracellular protease production among clinical isolates of *Staphylococcus aureus* due to different levels of expression of the protease repressor sarA. Infect Immun.

[41] Pizzaro-Cerda J, Cossart P (2006). Bacterial adhesion and entry into host cells. Cell.

[42] Tang J, Chen J, Li H, Zeng P, Li J (2013). Characterization of adhesin genes, staphylococcal nuclease, hemolysis and biofilm formation among *Staphylococcus aureus* strains isolated from different sources. Foodborne Pathog Dis.

[43] O'Neill E, Pozzi C, Houston P, Humphreys H, Robinson DA, Loughman A (2008). A novel *Staphylococcus aureus* biofilm phenotype mediated by the fibronectin-binding proteins, FnBPA and FnBPB. J Bacteriol.

[44] Mann EE, Rice KC, Boles BR, Endres JL, Ranjit D, Chandramohan L (2009). Modulation of eDNA release and degradation affects *Staphylococcus aureus* biofilm maturation. PLoS One.

[45] Saïd-Salim B, Dunman PM, McAleese FM, Macapagal D, Murphy E, McNamara PJ (2003). Global regulation of *Staphylococcus aureus* genes by Rot. J Bacteriol.

